# Results of a randomized controlled trial of level IIb preserving neck dissection in clinically node-negative squamous carcinoma of the oral cavity

**DOI:** 10.1186/s12957-018-1518-z

**Published:** 2018-11-08

**Authors:** Manoj Pandey, Senniappan Karthikeyan, Deepika Joshi, Mohan Kumar, Mridula Shukla

**Affiliations:** 1 Department of Surgical Oncology, Institute of Medical Sciences, Banaras Hindu University, Varanasi, 221 005 India; 2 Department of Neurology, Institute of Medical Sciences, Banaras Hindu University, Varanasi, 221 005 India; 3 Department of Pathology, Institute of Medical Sciences, Banaras Hindu University, Varanasi, 221 005 India; 4 SRL Religere, Lanka, Varanasi, 221005 India

## Abstract

**Background:**

The lymphatic spread from the cancers of the oral cavity follows an orderly progression and involvement of lower nodes without involvement of upper nodes and skip metastasis is rare. Selective neck dissections are increasingly being performed for node-positive patients; however, in node-negative patients the options of wait and watch, prophylactic radiotherapy, and prophylactic elective node dissections are debated. Quality of life and shoulder functions are important to choose the appropriate therapeutic modality.

**Patients and methods:**

Patients with oral squamous carcinoma with clinically and radiologically negative neck were randomized to IIb preserving superselective neck dissection or conventional supraomohyoid neck dissection. The primary end point of the study was recurrence of disease (clinical or radiological) and shoulder function as demonstrated by the clinical examination and electromyography. The secondary end point was quality of life as measured by the FACT-HN version 4 questionnaire at the end of 1 year follow-up.

**Results:**

The mean number of lymph node harvested per patient was 25.6 (range 8–85). Of the 32 patients, 3 had histologically positive node in level Ib, one of these patients had single positive node while the remaining two had three positive nodes in level Ib. At median follow-up of 36 months disease-free survival in IIb, sparing group was 83% compared to 91% in control arm, the difference in survival between two groups was statistically not significant (*p* = 0.694). EMG of the shoulder showed denervation pattern in 45% patients undergoing IIb preserving surgery at 1 month follow-up compared to 95% in conventional surgery group, this recovered in all patients but one at 3 months and 100% recovery was seen at 6 months.

**Conclusions:**

The results of the present study indicate that superselective IIb preserving neck dissections are technically feasible and appear to be oncologically safe procedures when performed as elective prophylactic procedures in highly select group of patients. A significant number of occult metastasis seen in the present study suggests prophylactic dissection to be better than wait and watch policy. Results also show initial higher shoulder morbidity at 1 month in patients undergoing IIb preserving dissections; however, at the end of 1 year recovery is complete and both procedures are comparable.

**Trial registration:**

The trial is registered at clinicaltrials.gov with registration no NCT00847717; registered on February 19, 2009.

## Introduction

Cancer of the oral cavity is the major health problem with over 2,70,000 new cases and 1,45,000 deaths reported worldwide annually [1]. Two third of this global burden is reported from low resource developing countries [2]. The cancer of the oral cavity has high propensity to disseminate along the lymphatics to cervical lymph nodes. Presence of metastatic disease in lymph nodes and extranodal spread are found to be independent predictor of survival [3, 4]. It is reported that patients with higher metastatic nodal burden have poor survival compared to those without nodal disease [3, 4].

It has also been reported that lymphatic spread from carcinoma of the oral cavity follows an orderly pattern of progression and risk of skip metastasis to lower nodes is negligible [5, 6]. With that in mind, the management of neck nodes has evolved considerably since the first report of radical neck dissection by Crile in 1906. Comprehensive functional and conservative neck dissections are routinely performed for neck positive disease; however, the management of clinically and radiologically negative neck is still controversial.

Elective prophylactic conservative neck dissection is practiced widely for node-negative disease due to the fact that neck dissection still remains the most accurate method of neck staging, thereby guiding adjuvant treatment and predicting the outcome [7–9]. Moreover, for malignancies requiring cervicofacial approach for extirpate the tumor, surgeons prefer to do a prophylactic neck dissection, as the neck will be entered anyway.

Wait and watch and delayed neck dissection, as and when the disease develop in neck is an option practiced for mainly the lesions that are excised intra orally [10]. Prophylectic neck irradiation is also practiced but remains controversial. As most resections for oral cancer require a cervicofacial approach with lip split for their excision especially in tobacco chewers with trismus, most of these patients undergo an elective prophylactic neck dissection.

One of the most frequent problems encountered in patients undergoing neck dissection is shoulder dysfunction [11–13]. This dysfunction is mainly due to injury to spinal accessory nerve that crosses level II and splits it into level IIa lying anterior to the nerve and level IIb that lies posterior-medially to the nerve, and nerve need be retracted to clear nodes in this area [11, 12, 14]. The second cause is injury to transverse cervical artery that traverses the level IV. As most node-negative patients undergo conservative neck dissection in form of supraomohyoid dissection arterial injury is usually not a cause for concern. However, the spinal accessory nerve is to be dissected and retracted to clear level IIb nodes that lie posterior to the nerve and hence is most important cause of shoulder dysfunction. As we know that the lymphatic spread follows an orderly pattern and involvement of IIb by skip metastasis is very rare in clinically and radiologically negative neck [5, 6], we hypothesized that in this subgroup of patients a IIb sparing neck dissection can safely be carried out. It is further hypothesized that in such a dissection the handling of the spinal accessory nerve is minimized and hence its injury and resulting shoulder dysfunction can be altogether avoided. We report here the results of randomized controlled trial comparing results of superselective IIb preserving neck dissection with comprehensive clearance of level I to III (supraomohyoid neck dissection) in node-negative squamous oral cancer.

## Material and methods

Between December 2007 and August 2009, 32 patients with oral squamous carcinoma with clinically and radiologically negative neck were randomized to IIb preserving superselective neck dissection or conventional supraomohyoid neck dissection. The trial was approved by institute ethics committee and written informed consent was obtained from all study participants. The study was single blinded as the participants did not know in advance as to which treatment will be offered to them, the randomization was done by KS. The inclusion and exclusion criteria were as follows.

### Inclusion criteria

Patients with clinically and radiologically negative neck, histologically proven squamous carcinoma of the oral cavity, over 18 years of age with ability to give consent.

### Exclusion criteria

Patients with synchronous primaries, distant metastasis, previous surgery on neck, previous radiotherapy, history of previous head and neck cancers except basal cell carcinoma, and pregnant and lactating women were excluded.

### Preoperative work-up and study design

Preoperative work-up included a detailed clinical examination followed by radiological evaluation of the neck using computerized tomography (CT) scan, after the biopsy confirmation of the primary tumor. Preoperative shoulder function was evaluated by a detailed clinical examination demonstrating range of shoulder abduction and flexion with goniometry and electromyography of the trapezius muscle. Quality of life was measured using FACT-HN version 4 questionnaire in native Hindi language.

### Operative procedures

In the control arm, a standard conservative neck dissection (supra omohyoid neck dissection) was carried out. After the dissection, all nodal stations were labeled separately and were sent for histopathology. In the treatment arm, the dissection in level II was stopped at the level of spinal accessory nerve. All the content of level IIb that lies posterior to the nerve was left undisturbed. All nodal stations were labeled before being sent for histopathology.

### Postoperative course and follow-up

Postoperative radiotherapy was given to patients with positive or close margin of excision of primary, T3 or T4 primary tumor, histologically positive lymph nodes or extracapsular spread if present or any other poor prognostic marker in primary like lymphovascular or perineural invasion. Patients were placed on regular monthly follow-up.

The primary end point of the study was recurrence of disease (clinical or radiological) and shoulder function as demonstrated by the clinical examination and electromyography. The secondary end point was quality of life as measured by the FACT-HN version 4 questionnaire at the end of 1 year follow-up.

At 1, 3, 6, and 12 months interval patients were evaluated by ultrasonography of the neck, evaluation of the shoulder function by clinical examination demonstrating range of movement by goniometry and EMG of trapezius muscle. At each of this evaluation, the quality of life was also recorded. Statistical analysis was carried out using Mann-Whitney *U* test, one-way ANOVA, and paired *t* test.

## Results

Of the 32 patients recruited, 12 (37.5%) were randomized to level IIb sparing neck dissection while 20 (62.5%) were randomized to control using computer-generated randomization. The patients’ characteristics of two groups are detailed in Table 1. The mean number of lymph node harvested per patient was 25.6 (range 8–85). The average number of nodes harvested per level in two groups is detailed in Table 2, while Table 3 describes the lymph node ratio (percentage). There was no statistical difference in the number of nodes harvested in two groups, and lymph node ratio was less than 5% in both groups. Of the 32 patients, 3 had histologically positive node, two of these patients had single positive node while the remaining had three positive nodes in.

**Table 1:** Patient characteristics

Variable	Sub group	2b sparing	Control	*P* value
Gender	Male	10	18	0.6
	Female	2	2	
Primary site	Buccal mucosa	8	10	0.15
	Lower alveolus	0	6	
	tongue	4	4	
Premalignant lesion	Leukoplakia	4	4	0.43
Habits	Tobacco chewing	8	17	0.16
	Chewing + smoking	2	3	
Clinical T stage	cT2	9	12	0.301
	cT3	2	2	
	cT4	0	5	
	cTx	1	1	
TNM stage	I	1	1	0.234
	II	8	12	
	III	3	2	
	IV	0	5	
Surgery for primary	WLE	12	20	
Bone resection	Hemi mandibulectomy	0	7	0.08
	Marginal mandibulectomy	0	1	
	Segmental mandibulectomy	0	2	
	Upper alveolectomy	1	0	
Reconstruction	Nasolabial	1	4	0.5
	Sternomastoid	2	2	
	SSG	3	2	
Neck dissection	I–III	11	19	
	I–V	1	1	
Pathological T	pT1	5	5	0.45
	pT2	4	7	
	pT3	1	1	
	pT4	1	6	
	pTx	1	1	
Margin	Close	3	4	0.5
	Negative	6	10	
	Positive	3	6	
Lymphovascular invasion	Present	2	7	0.263
Perineural invasion	Present	0	4	0.12
Radiotherapy	Yes	2	11	0.05
Chemotherapy	Yes	3	3	0.6
Recurrences	Yes	1	2	0.6

**Table 2:** Lymph node harvest

Level	IIb preserving		Control		*P* value
	Mean (*n*)	SD	Mean (*n*)	SD	
Ia	3	1.4	3.1	2.1	0.88
Ib	5.5	2.9	4.7	2.6	0.459
IIa	7.6	8.4	7.1	7.5	0.84
IIb	–	–	6.1	4.9	–
III	4.3	3.3	5.7	3.8	0.29

**Table 3:** Lymph node ratio

Level	IIb sparing			Control group		
	Harvested	Positive	Ratio (%)	Harvested	Positive	Ratio (%)
Level Ia	36	0	0	62	0	0
Level Ib	66	1	1.5	95	3	3.1
Level IIa	92	1	1	142	0	0
Level IIb	–	–	–	123	0	0
Level III	52	0	0	115	0	0
Total	259	2*	0.77	561	3*	0.53

*All patients were clinically N0, and only 3 patients had 5 positive nodes

### Loco-regional failure

Three patients had loco-regional failure in IIb sparing group. The failure occurred at primary site within a month of surgery in one of the patient despite a negative margin. This patient was treated with reexcision and concomitant chemo radiation and is disease-free at 118 months, while another developed a second primary after 98 months. The third recurrence occurred at 32 months and was treated with palliative chemotherapy, the patient died 4 months later. In the control group too there were three failures, one in primary site, one in neck, and the third patient had local recurrence with lung metastasis at 65 months. The patient with neck failure had multiple nodes in level II and III at 6 months of follow-up and was salvaged with a comprehensive neck dissection and is still alive at 118 months disease-free. One patient with local recurrence was salvaged with surgery and radiotherapy, and is also alive and disease-free, the third patient with metastasis died after 7 months.

At median follow-up of 36 months, the disease-free survival in IIb sparing group was 83% compared to 91% in control arm, the difference in survival between two groups was statistically not significant (*p* = 0.694).

### Shoulder function

The results of the shoulder function are detailed in Table 4. The results show significant restriction in shoulder abduction in control group at 1, 3, and 6 months of follow-up; however, the movements were comparable at 1 year. No difference in shoulder flexion was observed in either group.

**Table 4:** Function

	IIb sparing		Full dissection		Mann–Whitney *U* test	*p* value
	*n*	Mean ± SD	*n*	Mean ± SD		
Shoulder abduction						
Preop	12	160.54 ± 5.14	20	162.10 ± 5.17	86.500	0.330
1 month	12	146.36 ± 14.05	20	132.90 ± 12.08	54.000	**0.020**
3 months	2	151.54 ± 10.51	19	139.57 ± 10.68	32.500	**0.009**
6 months	9	155.86 ± 3.84	16	146.62 ± 9.08	20.000	**0.016**
12 months	7	156.66 ± 4.16	8	152.37 ± 6.75	7.000	0.304
18 months	3	157 ± 0.00	1	160 ± 0.00		
Shoulder flexion						
Preop	12	139.18 ± 4.46	20	140.30 ± 3.59	94.000	0.504
1 month	12	133.45 ± 6.45	20	130.85 ± 6.06	76.500	0.162
3 months	2	133.33 ± 5.56	19	132.94 ± 4.39	75.500	0.621
6 months	9	136.71 ± 4.49	16	134.87 ± 3.63	41.500	0.328
12 months	7	135.66 ± 3.21	8	134.87 ± 4.54	10.500	0.756
18 months	3	140 ± 0.00	1	132 ± 0.00		

Data in bold are significant *P* values

### Electromyography

EMG of the shoulder showed denervation pattern in 45% patients undergoing IIb preserving surgery at 1 month follow-up, this recovered in all patients but one at 3 months and 100% recovery was seen at 6 months. In the control group, denervation was seen in 95% patients at 1 month that showed full recovery at the end of 1 year except for one patient who kept showing denervation pattern (Table 5).

**Table 5:** EMG

Time		2b sparing	Control	*p* value
Pretreatment	Normal	12	20	
At 1 month	Normal	2	5	0.349
	Mild	5	4	
	Moderate	3	10	
	severe	2	1	
At 3 months	Normal	6	10	0.538
	Mild	3	4	
	Moderate	1	5	
	severe	2	1	
At 6 months	Normal	9	16	0.07
	Mild	0	4	
	Moderate	2	0	
	severe	1	0	
At 12 months	Normal	9	19	0.03
	Mild	3	1	
	Moderate	0	0	
	severe	0	0	
At 18 months	Normal	11	20	–
	Mild	1	0	
	Moderate	0	0	
	severe	0	0	

### Quality of life

No significant difference in quality of life was observed in two groups; however, patients undergoing IIb preserving dissections had better general well-being, physical well-being, head- and neck-specific scores, and total FACT-HN scores at 6 months of follow-up (Table 6).

**Table 6:** Quality of life

	Preop (mean ± SD)			1 month (mean ± SD)			3 months (mean ± SD)			6 months (mean ± SD)			12 months (mean ± SD)		
	IIb sparing	Full dissec	p* value	IIb sparing	Full dissec	p* value	IIb sparing	Full dissec	p* value	IIb sparing	Full dissec	p* value	IIb sparing	Full dissec	p* value
GP (general physical)	18.92 ± 2.27	18.70 ± 4.50	0.878	19.08 ± 4.64	17.70 ± 3.71	0.360	21.20 ± 4.75	18.42 ± 5.65	0.196	26.50 ± 1.19	22.50 ± 4.87	**0.034**	26.75 ± 1.89	24.30 ± 4.47	0.320
GS (general social/family)	20.00 ± 5.84	20.25 ± 4.33	0.891	21.67 ± 5.19	21.05 ± 3.25	0.681	21.90 ± 4.45	22.21 ± 3.13	0.828	24.0 ± 2.72	23.38 ± 2.55	0.586	24.75 ± 2.50	24.0 ± 2.62	0.634
GE (general emotional)	7.67 ± 5.78	11.40 ± 4.97	0.063	14.58 ± 5.97	14.50 ± 5.19	0.967	17.40 ± 5.46	15.63 ± 5.25	0.403	22.62 ± 1.06	18.62 ± 5.72	0.066	23.0 ± 0.816	20.10 ± 5.80	0.350
GF (general functional)	9.33 ± 6.31	12.1 ± 4.48	0.158	13.33 ± 6.32	11.95 ± 5.49	0.520	17.10 ± 6.29	13.36 ± 6.51	0.150	22.12 ± 2.41	18.62 ± 4.95	0.074	24.25 ± 1.25	20.60 ± 4.59	0.152
HN (head and neck specific)	32.17 ± 4.72	35.6 ± 2.32	**0.010**	28.58 ± 5.05	25.60 ± 4.96	0.113	32.80 ± 7.13	27.05 ± 6.23	**0.033**	36.38 ± 5.09	30.44 ± 6.74	**0.040**	37.0 ± 5.80	30.10 ± 7.09	0.112
Total	88.08 ± 11.35	98.05 ± 15.49	0.063	97.25 ± 21.60	90.80 ± 18.06	0.371	110.40 ± 23.62	96.68 ± 22.31	0.135	131.62 ± 10.46	113.56 ± 20.71	**0.031**	135.75 ± 10.81	119.10 ± 22.82	0.195

*p value by ANOVA test

Data in bold are significant *P* values

## Discussion

Node-negative oral cancer has perplexed surgeons and oncologist. With improvements in radio diagnosis and guided cytology technique coupled with studies detailing geographical distribution of neck nodes showing rarity of skip metastasis and the knowledge that elective dissection at the time of recurrence does not alter survival has made researchers think in terms of superselective neck dissections.

The results of the present study indicate that superselective IIb preserving neck dissections are technically feasible and oncologically safe procedures when performed as elective prophylactic procedures in select group of patients. Many other studies too have shown similar disease control and survival with selective or super selective neck dissections even in node-positive disease [7, 15–23].

The shoulder morbidity is one of the major concerns for prophylactic dissections [11–13]. Our results indicate significant shoulder abduction morbidity in patients undergoing classical conservative neck dissections. The results are supported by the EMG that showed denervation in 95% of the patients in this group. However, these morbidity recover with active physiotherapy within 1 year. No difference in shoulder flexion was observed in either group. Hence, in long term, there is no difference observed in shoulder function in either IIb sparing or classical conservative selective neck dissections. Similarly, the quality of life results also show no difference in quality of life in these patients again indicating no significant effect on shoulder function. Chan et al. [11] too have shown that the shoulder impairment is not significantly deranged at 1 year of follow-up. Lee et al. [24] suggested intraoperative monitoring to preserve the spinal accessory nerve; however, the present study indicate that this might not be necessary as the function after super selective neck dissections recovers to normal within 3 months of surgery. Similar results have also been reported by Goldstein et al. [13] who also showed improvement of shoulder function over time after conservative neck dissections. Giardiano et al. [12] showed significantly higher early shoulder morbidity with IIb dissections similar to our study; however, longer follow-up was not available in their series.

This is the first randomized controlled trial comparing the two conservative procedures in node-negative neck, though the sample size and inclusion of multiple sites is a limitation, warranting confirmation of results in further multicentric trial to validate the present results in different settings and different primary sites.

## Conclusion

The results of the present study indicate that super selective IIb sparing neck dissection is oncologically safe procedure with lower morbidity in node-negative squamous oral carcinoma patients. The procedure has no shoulder morbidity, and if the morbidity does occur, the recovery is much faster than the conventional selective neck dissection.
